# Modulation and Interaction of Immune-Associated Parameters with Antioxidant in the Immunocytes of Crab *Scylla paramamosain* Challenged with Lipopolysaccharides

**DOI:** 10.1155/2011/824962

**Published:** 2011-06-05

**Authors:** Singaram Gopalakrishnan, Fang-Yi Chen, Harikrishnan Thilagam, Kun Qiao, Wan-Fang Xu, Ke-Jian Wang

**Affiliations:** State Key Laboratory of Marine Environmental Science, College of Oceanography and Environmental Science, Xiamen University, Xiamen, Fujian 361005, China

## Abstract

Invertebrates are dependent on cellular and humoral immune defences against microbial infection. *Scylla paramamosain* is an important commercial species, but the fundamental knowledge on its immune defense related to the antioxidant and immune-associated reactions is still lacking. The study was to differentiate the responses of immune-associated parameters of haemolymph components in *S. paramamosain* when challenged with bacterial lipopolysaccharides (LPSs). The immunostimulating effects of LPS in crab by triggering various immune parameters (phagocytosis, lysozyme, antibacterial activity, phenoloxidase, and the generation of superoxide and nitric oxide) were investigated. Results showed that the generation of free radicals, phenoloxidase, lysozyme and antibacterial activities was significantly increased through the exposure periods. Conversely, total hemocyte count and lysosomal membrane stability decreased significantly as the exposure period extended to 96 h. The relationship between the antioxidant enzymes and immune reactions due to LPS was highly significant. In addition, ROS production was positively correlated with antioxidant showing immediate response of antioxidant defense to the oxyradicals generated. Overall, the study indicated that nonspecific immune components in hemocytes of crab showed active response to the LPS stimulation, and their responses suggested that many immune-associated parameters could be modulated and interrelated with the influence of antioxidants in crustaceans.

## 1. Introduction

Hemocytes play a fundamental role in invertebrate innate immune system [1] and its functional role includes phagocytosis of nonself molecule [2, 3]. NADPH-oxidase driven “respiratory burst” is characteristic of invertebrate phagocytes [4, 5], and the phagocytic defences are highly dependent on generation of superoxide anion and production of other reactive oxyradical species during the respiratory burst. In aerobic organisms, reactive oxygen species (ROS) can be continually generated in response to both external and internal stimuli [6], and the reactive oxygen intermediates produced during the process are highly toxic to microbes and recognized to have an important role in immune defense and could play multiple functions in many biological processes [7]. On the other hand, excess production of ROS could cause deleterious effects on biomolecules, and hence need to be scavenge by the cellular antioxidant defense system. 

Previous studies have reported ROS production, antioxidant enzyme defences and oxidative damage in invertebrates [8–10]. Importantly, recent research in crustaceans shows that ROS-dependent immunity is critical for host survival [11–13]; in addition, it has been reported that the antioxidant enzymes such as CAT and SOD could participate in crustaceans innate immune defense against immunostimulant [14–16]. Recently, a few studies have been undertaken on immunomodulation in crustaceans [17, 18]. However, little is known about the responses of these antioxidant enzymes (CAT and SOD) and their interaction with ROS production and other immune reaction in farmed crabs after the challenge of immune stimulant such as LPSs, which is an important component of Gram-negative bacterium. 

Our previous studies reported that antioxidant enzyme *Sp-*CAT and *Sp-*SOD gene expression were induced towards LPSs challenge in crab [19, 20], and it has been well documented that these antioxidant activities or their gene expression increased parallelly to immunostimulant challenge or pathogen infections in crustaceans [14, 16]. Following our previous work, the present study was designed to evaluate the hemocyte immune functions when the crab challenged with LPSs. The immune parameters analyzed include total hemocyte counts, membrane stability, phagocytosis, superoxide anion generation, nitric oxide production, phenoloxidase, lysozyme, and antibacterial activity. In addition, the relationship between the immune parameters with the antioxidant enzymes such as SOD and CAT was also analyzed. This investigation will provide general information on the immunomodulation of many immune-associated parameters and their interrelation with the antioxidants generated in *S. paramamosain* due to LPSs challenge.

## 2. Materials and Methods

### 2.1. Collection and Maintenance of Mud Crab *S. paramamosain*

*S. paramamosain* (300 ±  50 g in weight) purchased from a local commercial crab farm in Xiamen, Fujian Province, China were acclimatized at 25 ± 2°C for one week before the experiments were carried out.

### 2.2. Lipopolysaccharide (LPSs) Injection

LPSs from *E. coli* (L2880, Sigma, USA) was prepared as described previously [19, 20]. To study the immune parameters, 36 crabs were injected with a dose of 0.1 and 0.5 mg kg^−1^ LPSs, respectively, and the other 18 individuals were injected with an equal volume of sterile saline solution as control treatments. The crabs for each group (3 crabs per group) were separately reared in individual container under the same culture conditions. Meanwhile, three normal crabs were reared in an individual tank as a normal control group. Sampling was performed at different time intervals (3, 6, 12, 24, 48, and 96 h) after LPSs challenge. Haemolymph collection and separation of hemocytes and preparation of serum and hemocyte lysate suspension were described in detail in our earlier study [19, 20].

### 2.3. Total Hemocyte Count

Total hemocytes in haemolymph were determined by using hemocytometer. A sample of 20 *μ*L of diluted haemolymph was added to a hemocytometer and counted in microscope under 40x magnifications.

### 2.4. Superoxide Anion Production by Hemocytes

The generation of superoxide anion (O_2_^−^) by hemocytes of individual crab was quantified by measuring the reduction of nitroblue tetrazolium (NBT) following the procedure of Arumugam et al. [4]. Briefly, hemocyte suspension obtained from individual crab was incubated with 1 mg mL^−1^ of NBT at 22°C for 15 min. At the end of incubation, the reaction was stopped by adding 70% methanol and centrifuged (1500 rpm, 10 min, 4°C). Two mL of extraction fluid (6 mL KOH + 7 mL DMSO) were added to the pellet, to dissolve the insoluble formazan formed from NBT reduction. The samples were further centrifuged (8,000 rpm, 15 min, 4°C), and the O.D. was measured at 625 nm using spectrophotometer, against a reagent blank.

### 2.5. Nitrite Production

Nitric oxide (NO) production by crab hemocyte lysate was evaluated as described previously [21] by the Griess reaction, which quantifies the nitrite (NO_2_^−^) content of supernatants. Aliquots of HLS was incubated for 10 min in the dark with 1% (w/v) sulphanilamide in 5% H_3_PO_4_ and 0.1% (w/v) N-(1-naphthy)-ethylenediamine dihydrochloride. The O.D. of the samples was measured at 540 nm in a spectrophotometer against a suitable reagent blank. The molar concentration of nitrite in the samples was determined from a standard curve generated using known concentrations of sodium nitrite and was represented as *μ*M nitrite. 

### 2.6. Detection of Phenoloxidase (PO)

Plasma PO was investigated following the procedure of Asokan et al. [22]. Briefly, plasma samples (100 *μ*L) were preincubated with the same volume of L-dihydroxyphenyl-alanine (L-DOPA) or with TBS for 20 min at 22°C. All incubation experiments were performed in the dark. The O.D. of both control and experimental was measured at 460 nm. Plasma samples were estimated for protein content by Bradford [23].

### 2.7. *In Vitro* Phagocytosis

Suspensions of lyophilised *Saccharomyces cerevisia *were prepared in a sterile saline solution (0.15 M NaCl). The cells were washed three times with saline solution and suspended (10^6^ cells mL^−1^) in 0.45 M NaCl. Hemocyte monolayers were prepared on glass slides, allowing the cells to attach for 15–20 min at 20°C. The monolayers were carefully rinsed with TBS and incubated with suspension of *S. cerevisiae *for 1 h at room temperature. After incubation, the monolayers were carefully rinsed with TBS, fixed for 10 min in methanol, stained with Giemsa for 20 min, and rinsed with distilled water [24]. Then, the slides were mounted and examined under the light microscope to record the phagocytosis of the yeast by the hemocytes.

### 2.8. Lysozyme Assay

Serum lysozyme assay was determined by using modified turbidometric assay developed by Hutchinson and Manning [25]. Briefly, 0.3 mg mL^−1^ suspension of freeze-dried *Micrococcus lysodeikticus *was prepared in 0.05 M Na_2_HPO_4_ buffer immediately before use and the pH adjusted to 6.0 using 1.0 M NaOH. Ten microlitres of serum were added to 250 *μ*L of the bacterial suspension and allowed to equilibrate at 28°C. Hen egg white lysozyme (HEWL), with a specified activity of 46 200 Units mg^−1^ was used as an external standard. The reduction in O.D. at 450 nm was determined over a 10 min period at 28°C in a microplate reader. The standard curve was constructed by using HEWL. The amount of lysozyme present in the serum was calculated from the standard.

### 2.9. Lysosomal Membrane Stability

Haemolymph (100 *μ*L) sample pipetted into 0.5 mL centrifuge tube and aliquots (10 *μ*L) of 0.33% neutral red (Sigma) solution in TBS was added to each tube, and the tube was incubated for 1 h at 10°C. The tubes were then centrifuged for 5 min and washed twice in TBS. Aliquots (100 *μ*L) of 1% acetic acid in 50% ethanol were added to all tubes. The tubes were covered with foil, incubated for 15 min at 20°C, and then read at 550 nm. 

### 2.10. Antibacterial Activity of Haemolymph

Antibacterial activity of the haemolymph was investigated by measurement of growth inhibition by turbidometry [26]. Briefly, 100 *μ*L of serum from both control and experimental groups were added to 96 wells plate. A log phase broth culture of *Aeromonas hydrophilla* suspension (100 *μ*L) in NB was prepared (~10^8^ bacteria mL^−1^; OD 600 = 0.509) and added to each of the experimental and control wells. Positive control with broth and bacteria were also maintained. Aliquots of 100 *μ*L sterile TBS and 100 *μ*L sterile broth were added to a well to act as a blank. The plate was incubated at room temperature and absorbance measured after 0, 1, and 24 h, respectively.

### 2.11. Statistical Analyses

The SPSS software version 11.0 for Windows was used for the statistical analysis. Results are reported as mean ± S.D. of three individuals per group per time point (*n* = 3). The data were processed by two-way analysis of variance (ANOVA) followed by Tukey's multiple-comparison post hoc test to identify statistical differences. Pearson correlation coefficient was used in the correlation matrix. Differences were statistically significant when *P* < .05 and  .01.

## 3. Results and Discussion

### 3.1. Total Hemocyte Count (THC)

**Figure 1:**
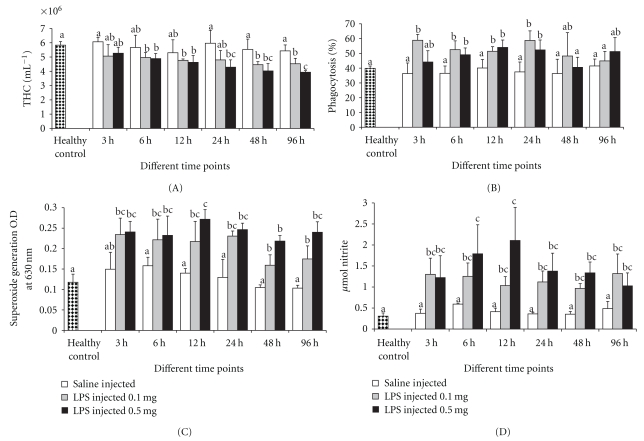
The effect of LPSs on (A) THC, (B) percent phagocytosis, (C) superoxide anion generation, and (D) nitric oxide. Data, representing the mean ± S.D. of 3 determinations using samples from different preparations, were analyzed using ANOVA followed by the Tukey post hoc test. The same letters (a, b, and c) indicate no significant difference between challenge groups at different exposure periods, whereas different letters indicate statistically significant differences (*P* ≤ .05) between different exposure periods and groups.

THC plays an important role in regulating the physiological functions including haemolymph coagulation, phagocytosis, encapsulation, and confinement of invasive organisms. In addition, it also involves in hardening of exoskeleton, carbohydrate metabolism, and transport or storage of protein and amino acid [27, 28], and more important THC will reflect the health status of the host. There was no significant difference in THC for crabs challenged with LPSs up to 24 h with the respective control groups; however, after 48 h, the THC decreased significantly in both groups challenged with LPSs (Figure 1(A)). Similar decrease in THC due to nonself challenge in crustaceans was described previously, which led to the rapid reduction in the numbers of circulating hemocytes [29–31]. Variations in hemocyte numbers may result in different defense activities including hemocyte migration to the injection site and hemocyte lysis [32]. The loss of hemocytes from the haemolymph might result from degranulation, lysis, and the formation of cell clumps or nodules [30].

### 3.2. Phagocytosis

 Phagocytosis is one of the most important parameter associated with immune reaction in both invertebrates and vertebrates [24, 33], which normally mobilizes lysosomes for the invading phagocytosed materials [34]. The effect of endotoxin on the phagocytic ability of the hemocytes of *S. paramamosain* is shown in Figure 1(B). After LPSs injection, the phagocytic ability of hemocytes of the crab was triggered significantly in both groups. Though increase in phagocytosis was observed after LPSs challenge in both groups, such increase in activity was significant only after 3 h and at 24 h after challenge. The similar result was observed in crustacean hemocytes as described previously, as the phagocytic activity triggered by LPSs was able to accelerate the cellular reactions [35].

### 3.3. Superoxide Anion Generation

Phagocytosis is also associated with the production of ROS namely superoxide anion generation (O_2_^−^) which is highly microbicidal [36]. Although ROS play an important role in host defense, over-induced and residual ROS may lead to cellular damage in the host. Consequently, phagocytosis becomes essential for cells exerting proper functions to rapidly eliminate excessive ROS, and thus maintain the homeostasis of organisms. This cellular reaction is immunologically vital and studied in crustaceans [18, 37]. Significant induction of superoxide generation observed following LPSs challenge in both groups indicated that the bacterial endotoxin can stimulate generation of superoxide in *S. paramamosain* even at a low dose (Figure 1(C)). These results were similar to those of Song and Hsieh [38] in which the relative *in vitro *intracellular O_2_^−^ production was enhanced twice for *Penaeus monodon *hemocytes treated by heat killed *Vibrio vulnificus*, *β*-1,3-1,6-glucan and zymosan. However, whether such induction would help crabs survival for a longer time if the crabs suffered from a pathogen remains to be further elucidated.

### 3.4. Nitric Oxide Generation

LPSs injection also stimulated an increased level of nitric oxide, a molecule considered as precursor of a variety of reactive nitrogen intermediates. As shown in Figure 1(D), there was up to 3- and 4-fold increase in nitrite generation in the case of hemocytes collected from LPSs-injected crab, and this increase was significant during the exposure period. The maximum nitrite generation was observed after 12 h after injection of 0.5 mg of LPSs to crab. As observed in crayfish *Procambarus clarkii* by Yeh et al. [39], the hemocyte-derived NO increased by two-fold following LPSs challenge.

### 3.5. Phenoloxidase Activity

**Figure 2:**
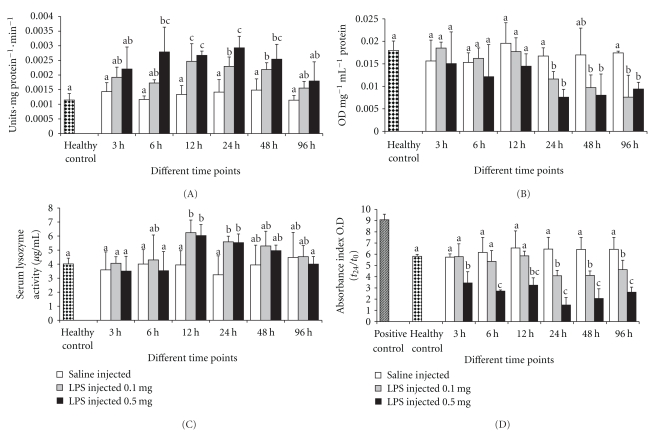
The effect of LPSs on (A) phenoloxidase, (B) membrane stability, (C) lysozyme activity, and (D) antibacterial activity. Data, representing the mean ± S.D. of 3 determinations using samples from different preparations, were analyzed using ANOVA followed by the Tukey post hoc test. The same letters (a, b, and c) indicate no significant difference between challenge groups at different exposure periods, whereas different letters indicate statistically significant differences (*P* ≤ .05) between different exposure periods and groups.

Phenoloxidase (PO) is an important humoral defense component in crustaceans, as it can be activated by nonself material. Its activation results in induction of a number of potent bioactive products which assist phagocytosis, cell adhesion, and formation of melanin deposits [40]. In general, PO is present in crab plasma in an inactive proPO state. Modulations in the levels of this important defense enzyme will have positively influence on the survivability of animals upon challenge with infectious pathogens. Earlier studies showed that LPSs induce prophenoloxidase activation and melanisation reactions [31, 41]. Significant induction of plasma PO due to endotoxin was observed when the crab was injected with LPSs, the levels of PO activity in the plasma were altered in both groups of LPSs-injected crabs, indicating active responses of proPO to a nonself molecule (Figure 2(A)). Earlier research on crustaceans' species demonstrated that PO activation by glucans or other nonself molecules generates a range of immunoactive agents and activities, including peroxinectin and ROS [42].

### 3.6. Lysosomal Membrane Stability

Stability of lysosomal membranes in hemocytes of *S. paramamosain *following LPSs injection was evaluated by the neutral red retention assay. Membrane stability is a sensitive indicator of lipid membrane integrity in shellfish [43]. Membrane stability can be affected by both chemical and nonchemical stressors, suggesting that hemocyte viability itself is central to the response to both immunological challenge and other stressors [44]. The results of the present study indicated that lysosomal membrane stability was significantly reduced after 24 h of LPSs challenge when compared to respective control groups (Figure 2(B)). With the challenge time increased, the membrane stability became weaker and the figure clearly showed that LPSs affected the lysosomal membrane. The stability of hemocyte lysosomal membranes following LPSs injection decreased and well correlated with the decrease of THCs. The similar phenomenon was founded in other studies [45].

### 3.7. Lysozyme Activity

Lysozyme is a lysosomal enzyme secreted by the host during phagocytosis and has an important bacteriolytic characteristic in the immune system of crustaceans against pathogenic bacteria. In the study, significant induction of lysozyme activity was only observed after 12 and 24 h post challenge in both groups (Figure 2(C)). An increase in phagocytic activity observed in *S. paramamosain* after LPSs injection reflected the involvement of induced lysozyme activity. In addition, the lysozyme activity began to raise after 12 h LPSs injection, which suggests that the cellular reactions are in the first line of defense followed by an increase in antimicrobial activity [35]. The present results were consistent with previous study in which lysozyme activity in the shrimp *Fenneropenaeus chinensis* increased by nonself molecule [46].

### 3.8. Antibacterial Activity

Earlier reports revealed that crustacean haemolymph has the ability to inhibit bacterial growth [39, 47, 48]. Yeh et al. [39] reported that hemocyte-derived NO promotes bacterial adhesion to hemocytes and increase the bactericidal activity of hemocytes. Bacterial growth was significantly slower in serum of LPSs challenged crab, indicating the antibacterial activity in serum was triggered due to endotoxin (Figure 2(D)). It was noted that the results were accompanying to the decrease in lysosomal membrane stability after 24 h LPSs injection in both groups. Similar findings were observed for the Chinese shrimp hemocytes as a response to injection with *Vibrio anguillarum* [49]. However, the high dose of LPSs injection suppresses the bacterial growth in all the exposure periods. 

### 3.9. Correlation Analyses and Summary

In earlier study, we reported that the gene expression of antioxidants such as SOD and CAT and their activities were induced significantly when the crab were challenged with LPSs [19, 20]; correspondingly, it was found in the present study that there was a relationship between the immune associated parameters and the antioxidants (Table 1). The correlation between the antioxidant and immune parameters such as phenoloxidase, antibacterial activity, phagocytosis, superoxide generation, and nitric oxide production was much high. In higher invertebrate species like arthropods and molluscs, NADPH-oxidase and phenoloxidase are two redox-catalyzing systems which play important physiological roles including immunological functions [4, 50, 51]. Though these two enzymes play different roles, the common function is the generation of superoxide radicals (NADPH-oxidase) and by lesser extent in producing phenoloxidase. 

Moreover, these two enzymes remain in an inactive state in the cytosol and are activated by various stimulants [5, 52]. Upon stimulation, phenoloxidase is activated, catalyzes the melanin synthesis, and deposits near the pathogen, and this cascade reaction is characterized by numerous redox intermediates which generate the superoxide [53, 54]. On the other hand, during phagocytosis, there will be an increase in production of ROS as a positive immune reaction by the host to invade the pathogen. It has been reported that nonself induced nitric oxide generation involving nitric oxide synthase in crustacean hemocytes and the generation of nitric oxide during phagocytosis as a cellular immune reaction [18]. In the immune system of higher invertebrates like arthropods and molluscs, lysozyme is one of the most important bacteriolytic agents against corresponding species of Gram-positive and Gram-negative bacteria, and during phagocytosis, the hemocytes produce lysozyme which actively participate in the inactivation of invading pathogens. In addition, the breakage of membrane of hemocyte might have increased acid phosphates activity which contributed in lysis and eventual decomposition of pathogens and by the evident of antibacterial activity shown to increase in the present study. All these cellular defense reactions were vital in immune response and have been well characterized in invertebrates.

 The correlation between antioxidant enzyme and oxyradicals produced were high in the present study. Increase in these oxyradicals can cause direct or indirect damage to the membrane and DNA. SOD is the first enzyme to deal with oxyradicals by accelerating the dismutation of superoxide generated, and CAT is a peroxisomal haemoprotein which catalyses the removal of H_2_O_2_ formed during the reaction catalyzed by SOD. ROS, thus, generated may modulate the levels of SOD, which leads to alteration of CAT activity as a chain reaction. In this sense, the antioxidants interact with excess oxyradicals produced in aerobic animals. Hence, the net result of these antioxidant enzymes provides a postphagocytosis self-protection in the host. Correlation between the free radicals generated in hemocytes and the antioxidants produced to counteract the free radicals were significantly high indicating that the antioxidant enzymes (SOD and CAT) detoxify the free radicals generated and these enzymes might act as important acute defense molecules under stress of immunostimulant challenge or microbial infection.

The present study showed that LPSs could modulate the immune parameters of the crab, and the response was quick, time dependent/dose dependent. The immune parameters such as phagocytosis, phenoloxidase, lysozyme, and antibacterial activity were all significantly induced after LPSs after injection indicating that crabs respond positively to LPSs stimulation. The overall results indicated that nonspecific components actively responded to the bacterial endotoxic stimulation and their responses implied that the immune-related parameters were involved in the immunomodulation of crustacean's immune system. Furthermore, the correlation existing between the immune associated parameters and antioxidants strongly supports our previous work that the antioxidant enzymes play an important role in ROS detoxification in host-microbe interactions. Future work will be focus on evaluating whether the induced antioxidant and immune-associated reactions provoked by LPSs will together enhance the capability of *S. paramamosain* resistant to an infection caused by live pathogenic bacteria.

## Figures and Tables

**Table 1:** Correlation matrix for measured parameters in *Scylla paramamosain* challenged with LPSs.

Correlation Matrix												
	LMS	PO	LY	AB	PHA	SO_2_^−^	NO	THC	HLS-SOD	HLS-CAT	SERUM-SOD	SERUM-CAT
LMS	1.000	−0.483*	−0.293	0.807**	−0.202	−0.374	−0.453	0.684**	−0.326	−0.441	0.192	0.265
PO		1.000	0.565*	−0.799**	0.606**	0.787**	0.799**	−0.601**	0.652**	0.671**	0.249	0.298
LY			1.000	−0.313	0.540*	0.399	0.444	−0.549∗	0.069	0.432	0.000	0.066
AB				1.000	−0.416	−0.733**	−0.724**	0.724**	−0.673**	−0.671**	−0.144	−0.117
PHA					1.000	0.769**	0.693**	−0.520*	0.433	0.661**	0.206	0.381
SO_2_^−^						1.000	0.863**	−0.559*	0.725**	0.818**	0.394	0.561*
NO							1.000	−0.590**	0.709**	0.726**	0.343	0.304
THC								1.000	−0.446	−0.632**	0.125	−0.006
HLS-SOD									1.000	0.742**	0.690**	0.638**
HLS-CAT										1.000	0.472*	0.639**
SERUM-SOD											1.000	0.772**
SERUM-CAT												1.000

**Correlation is significant at the 0.01 level (2-tailed); *Correlation is significant at the 0.05 level (2-tailed).

LMS: lysosomal membrane stability, PO: phenoloxidase, LY: lysozyme activity, AB: antibacterial activity, PHA: phagocytosis, SO_2_^−^: superoxide anion generation, NO: nitric oxide, THC: total hemocyte count, HLS: hemocyte lysate solution, SOD: superoxide dismutase, and CAT: catalase.
